# A Compact Forearm Crutch Based on Force Sensors for Aided Gait: Reliability and Validity

**DOI:** 10.3390/s16060925

**Published:** 2016-06-21

**Authors:** Gema Chamorro-Moriana, José Luis Sevillano, Carmen Ridao-Fernández

**Affiliations:** 1 Department of Physiotherapy, University of Seville, Sevilla 41009, Spain; mcrf.2817@gmail.com; 2 Department of Computer Technology and Architecture, University of Seville, Sevilla 41012, Spain; sevi@atc.us.es

**Keywords:** walking, crutches, instrumentation, validity, reliability

## Abstract

Frequently, patients who suffer injuries in some lower member require forearm crutches in order to partially unload weight-bearing. These lesions cause pain in lower limb unloading and their progression should be controlled objectively to avoid significant errors in accuracy and, consequently, complications and after effects in lesions. The design of a new and feasible tool that allows us to control and improve the accuracy of loads exerted on crutches during aided gait is necessary, so as to unburden the lower limbs. In this paper, we describe such a system based on a force sensor, which we have named the GCH System 2.0. Furthermore, we determine the validity and reliability of measurements obtained using this tool via a comparison with the validated AMTI (Advanced Mechanical Technology, Inc., Watertown, MA, USA) OR6-7-2000 Platform. An intra-class correlation coefficient demonstrated excellent agreement between the AMTI Platform and the GCH System. A regression line to determine the predictive ability of the GCH system towards the AMTI Platform was found, which obtained a precision of 99.3%. A detailed statistical analysis is presented for all the measurements and also segregated for several requested loads on the crutches (10%, 25% and 50% of body weight). Our results show that our system, designed for assessing loads exerted by patients on forearm crutches during assisted gait, provides valid and reliable measurements of loads.

## 1. Introduction

Gait training is one of the most prominent processes in the physiotherapy area, as gait is one of the main functions of human beings [1,2,3]. Frequently, recovery of musculoskeletal injuries to an affected lower member involves gait training using forearm crutches for partial unloading thereof [2,3,4]. In this sense, the current trend is to load the maximum amount of weight depending on the lesion and its evolution on the lower limb. Authors such as Xu *et al.* [5] assert that lower limb unloading damages segmental circulation and decreases muscle tone, which consequently reduces the osteoblastic action and increases osteoclastic action. This result particularly affects the recovery of patients with sequelae of fractures in lower members, even more than in those who suffer from osteopenia or osteoporosis [6]. If we add inhibition of joint and muscle plantar proprioceptive receptors to these aspects, a functional deficit is obtained in the patient that could hamper and delay their recovery [7,8]. On the other hand, an excessive load on the injured lower member can lead to compressions or undue stress of structures on the patient even without regeneration or in the process of recovery, thus causing relapses and sequelae of their original injury [9].

Therefore, it is fundamental that there are feasible measuring instruments in daily practice that objectively monitor or control the unloading that the patients perform on an injured limb. The design of this kind of measuring instrument is an area of active research. As force platforms are usually very expensive and limit user movements, other devices are more convenient. For instance, Gonzalez *et al.* [10] have recently developed a system for gait monitoring based on a wireless sensorized insole. However, although sensorized insoles allow us to identify foot pathologies [11], they have several drawbacks when used during aided gait: for instance, they have to be adapted to each specific user (e.g., his/her foot size) and require using shoes (which may not be used with bandages). On the other hand, crutches can be shared by different users and used in many different situations without any modifications. Furthermore, they permit the comparison of ipsilateral and contralateral loads. One of the authors (Chamorro-Moriana) as part of her PhD Thesis, designed the so-called GCH System 1.0 (GCH is the abbreviation of the name of its inventor) that allows measuring loads applied on the forearm crutches, a prototype patented in 2009 [12], developed and validated in a previous study [13].

Other authors have also recently discussed the use of crutches for gait monitoring, with a prototype and some preliminary results being reported in another study [14]. However, their system overlooks many clinical issues. For instance, it includes a vibrating signal in the crutch grip that is activated only if the patient loads more weight on the crutch than recommended. Clinically, this is wrong feedback since the most important problem for the patient occurs when the patient loads less than the appropriate amount on the crutch though their arm, thus implying an excessive and dangerous load on the affected lower limb. To the best of our knowledge, none of the systems described in literature (including [13,14]) are ready to be used in clinical settings.

The system described in this paper, which we name GCH System 2.0, is an improved version of GCH System 1.0 described in another study [13]. The use of GCH System 1.0 in the laboratory enabled us to verify its effectiveness, after an extensive trial period, and also take into account the feedback provided by real users and the researchers themselves, and a number of improvements were introduced in the original design that led to GCH 2.0. The aim of this paper is to describe all of these improvements, as well as the design, validation and calculation of the reliability of the GCH 2.0 load measurement system. In addition to these technological and methodological contributions, specific clinical features were added that let the new system adapt to different processes of the functional recovery, as the authors show in the following section. The new tool will allow us to objectively know the weight bearing exerted by patients on crutches during the aided gait, and, thus, the unloading on the affected lower limb. In addition, it will enable training based on the patient’s biofeedback [15,16]. Finally it will promote monitoring, evaluation and analysis of load progressions to establish clinical protocols [17].

The rest of the paper is organized as follows: Section 2 describes the GCH 2.0 system, with an in-depth comparison to the first GCH prototype. Section 2 also describes the experimental setting of the study of the validity and reliability of measurements via a comparison with the validated AMTI OR6-7-2000 platform. Section 3 presents the results of these experiments, which are discussed in Section 4. Finally, Section 5 presents our Conclusions.

## 2. Experimental Section

### 2.1. GCH System 2.0

In this section, the main components of GCH 2.0 are described, emphasizing the main improvements carried out on the previous prototype [13].

The core of GCH 2.0 System consists of the coupling of a miniature force sensor, an Exact Sensor Instrument’s EX601D (Shenzhen Exact Sensor Instrument Co., Ltd., Shenzhen, China) [18], within the distal part of the forearm crutch shaft. Compression load cell features include: cylindrical (in the shape of a coin), diameter of 19 mm, stainless steel measuring element, hermetically sealed (fully welded) and easy installation. Safe load limit = 100 kg, rated output = 0.7198 mV/V, linearity ≤0.05% Full Scale (FS), hysteresis ≤0.05% FS, repeatability ≤0.03% FS, Zero balance ±2% FS and operating temperature range = −20 °C to 60 °C.

The said cell is connected to the electronic board and power batteries housed in this area. The data acquisition card has the function of emitting a radiofrequency signal. The communication protocol used is SimpliciTI, a low-power Radio Frequency (RF) protocol from Texas Instruments Inc. (Dallas, Texas, USA) [19] at 898 MHz with a period of 80 samples per second. The digital and amplified signal arrives at an ultra low power MSP430 microcontroller also from Texas Instruments (input voltage 2.4 V DC—Direct Current, battery/autonomy 6000 mAh).

**Figure 1:**
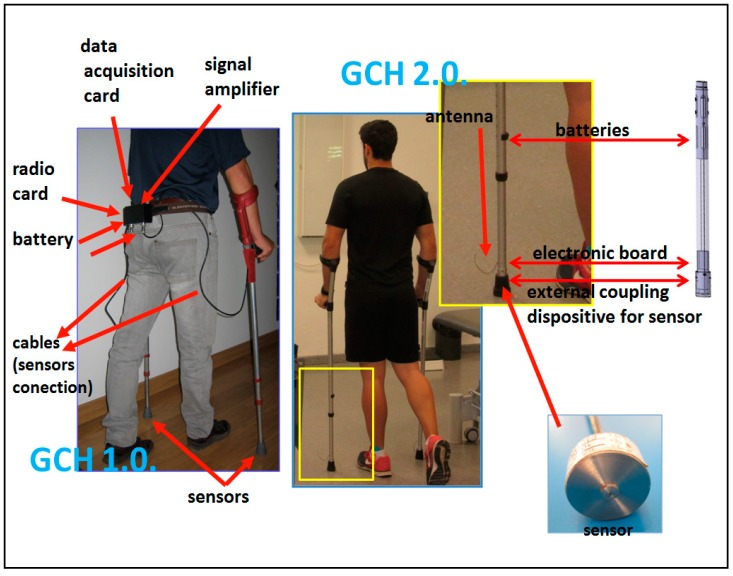
Comparison of GCH 1.0 and GCH 2.0.

GCH 2.0, as opposed to GCH 1.0 [12], introduces a compact design of the system integrating all these elements inside the crutch shaft. The sensor with its coupling mechanism; the data acquisition and radio cards, incorporated in a single printed circuit board (PCB); and the power supply are integrated in the most distal part of the crutch, an independent and extensible pipe to allow for the height adjustment tube (Figure 1). All external cables are eliminated. Manufacture of the PCB has been conducted with miniature electronic components, as a surface mount device (SMD). This allows us to integrate electronics inside the crutch, unlike the first prototype that wired the sensor installed inside the crutch with an external box that patients had to carry in their belt.

It is worth noting that when the GCH 2.0 crutch is started, the offset process is automatically activated. Once the crutch is on, it is placed vertically on the floor for 5 s, thus its weight is not recorded. This method allows us to exchange or add the usual standard components to the crutches (ergonomic handles, casings, beads, *etc.*) without the weight difference affecting measurements.

One of our main objectives with this new system is to provide a compact and ready-to-use device that could be used not only in clinical trials or under direct supervision by the physiotherapist, but also during daily life, thus speeding up the patient’s recovery. With this aim in mind, a clock-shaped, portable receiver has been introduced that can be used independently by the patient while performing the aided gait with crutches even outdoors. Thus, not only would they walk on stable ground within a room, but they could also walk on steps, ramps or uneven floors, *i.e.*, the usual outdoor obstacles and difficulties [20]. In addition, thanks to the use of a compact force sensor, GCH 2.0 does not require frequent calibrations.

Regarding the batteries, we decided to replace them with double standard AA that, together with an adapter, were also integrated inside the crutch, with a minimum amperage of 3000 mA each to prolong their use. The batteries are directly rechargeable through a connector or by extracting them and using a standard charger. This latter mode allows the user to replace the batteries while the used ones are being charged so that the crutches are available at all times.

The design of a specific program has been another major improvement in the applicability of GCH 2.0 as a measurement tool. The Crossbow sensor of GCH System 1.0 emitted signals that were monitored and recorded by a generic program called Moteview 2.0 (Moog Crossbow, East Aurora, NY, USA) [21]. This program works at a frequency of 0 to 10 Hz, so that even using the maximum frequency, the curve recorded was not accurate enough. The new GCH 2.0 software (called GCH Control Software 1.0) records data up to 80 Hz, thus obtaining a more optimized curve of weight bearing at each step. The basic application covers one or two modules (one for each crutch), although the system is able to cover more. That is, many patients can be walking with instrumented crutches at the same time. The signal sent by the crutches is detected by a small USB receiver that is connected to a computer (“fixed system”) by means of a virtual COM (Communication) port, or by a receptor incorporated into a watch, mobile phone, pendant or substitute (the “portable system” described before). This portable receiver provides autonomy to the patient in order to perform aided gait outdoors.

The fixed system is used during patient care in the clinic or laboratory as a training or research object, respectively. The software translates the millivolts from the sensor signal into units of force (force kilograms—kg), records the data, analyzes them and allows their numerical and graphical display of the loads that the subject carries out on screen in real-time. Besides specific graphs of weight bearing by each crutch, the unification or overlapping of both are obtained. These graphs can be seen by the patient through a canon projector to perform a visual feedback during gait.

This visual feedback is part of what we call a “self-correcting” mechanism. Thanks to this mechanism, new in GCH 2.0, the patient receives acoustic and/or visual information about the load exerted on the crutches. The visual feedback is based on the above-mentioned projection on a screen of the amount of load exerted, which is directly proportional to the injured lower member unloading. This feature is mainly intended for patient’s training during physiotherapy sessions. On the other hand, the acoustic feedback allows the system to be used without supervision even outdoors. With this self-correcting mechanism, if the amount of load is wrong, the subject is able to increase or reduce the force to improve the accuracy and exactly manage the ideal load recommended by the physiotherapist.

Finally, the program contains a database specifically designed for patients, in which we can record all their data and the applied sessions with our tool. Likewise, the recordings of the loads applied, number of steps, mistakes made, *etc.*, are designed for their posterior analysis within the care or research area.

The System was registered at the Spanish Patent and Trademark Office with number P201031779, and international expansion has been carried out [22]. A summary of all the improvements is shown in Table 1.

#### Basic Functions of the GCH System 2.0

**Figure 2:**
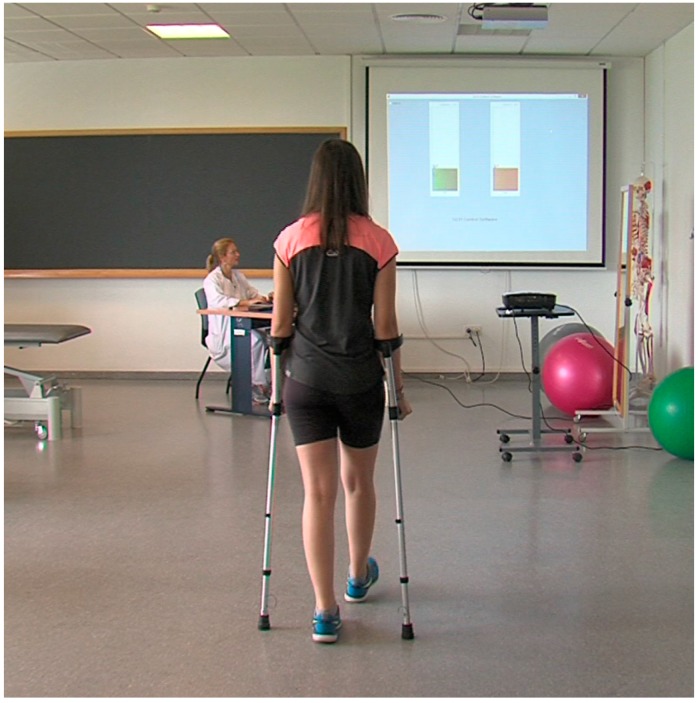
Individual walking while the quantity of the load exerted on the crutches is observed on the screen to improve its accuracy. The image on the board, which is different from the computer screen, is a specific chart for the patient.

From an operational point of view, the two main functions of GCH 2.0 are:

- Load control. The objective measurement of the loads applied to the crutches is the basis of the System. It shows the kilograms exerted on the crutches and the percentage of patient body weight (PPBW). This datum is the most relevant in the clinic, which always requires the subject’s current weight to be entered. The percentages allow researchers to compare intra-subject and inter-subject tests in order to establish treatment protocols.
- Feedback mechanism. The feedback information includes, individually, the ideal load exerted on the crutches (directly proportional to the unloading on the injured lower member), PPBW, according to the pathology and the treatment phase, introduced into the software as well as a percentage of error tolerated clinically due to excess or defect load. The fixed system alerts the subject of the mistakes made during gait for immediate self-correction (Figure 2). Acoustic signals, a continuous whistling, will be used if the recommended load is exceeded or discontinuous if it does not reach it; and visual, by using a projector. The portable system only uses an audible feedback. The fixed system shows only the feedback information useful for the patient’s training in the projector, and additional personalized clinical information (useful for the physiotherapist/researcher) on the computer screen.

The inclusion of the patient’s weight especially benefits the functionality of the program, since it allows us to extrapolate load amounts to PPBW [9], both in the recommendation of the ideal weight as well as the margins of errors allowed. This offers the possibility of performing intra-subject and inter-subject comparisons. Only in this way can we advance towards the creation of protocols of performance based on scientific evidence [24].

### 2.2. Study Design

The research presented in this section is a concordance study on the equivalence between the values obtained for the same variable (vertical reaction force), under the same conditions and synchronously by two different measurement procedures [25,26]: the force measurement system applied to a crutch, GCH System 2.0, and the already validated AMTI OR6-7-2000 force platform. The following sections, 2.3 to 2.5, present sufficient details to allow reproducibility of the results.

### 2.3. Study Variables

The variables analyzed were quantitative, since they referred to amounts of load or the difference between various measurements.

- Variable 1: GCH. Vertical reaction force of the Platform on the crutch (*Z* component) measured using our System. This variable is secreted in GCH_right and GCH_left, right and left crutches, respectively. The kg is used as a unit of measurement for GCH.
- Variable 2: Platform (kg). Vertical reaction force of the crutches on the Platform (*Z* component) calculated by the AMTI Platform. The unit of measurement was the kg.

We only consider the axial force or *Z* component because, clinically, we are interested in analyzing the peak load and its maintenance over time, and this peak load occurs when the crutch is perpendicular to the ground. At present, the components *X* and *Y* are negligible, being assumed as measurement errors.

### 2.4. Measurements and Participants

The measurements were made by means of the GCH System 2.0 and the AMTI Platform during the assisted gait carried out by 30 participants, 18 women (66.7%) and 12 men (33.3%), with an age range from 18 to 45 years (mean = 29.87 years; Standard Deviation SD = 7.26).

Different PPBW applied to the crutches were requested in order to obtain heterogeneous measurements during gait. The sampling was considered non-probabilistic due to guidelines provided. We obtained loads between 2.13 kg and 50.60 kg.

For each crutch (2) and participant (30), nine measurements were taken. Of these, three belonged to a requested load on the crutch at 10% of body weight, another three at 25% and three more at 50% or the maximum possible. A total of 540 measurements were taken (270 with each crutch). These values were compared with the 540 measurements undertaken by the force platform. The number of measurements (540) is high enough according to the required sample size when comparing two means in two samples [27].

Finally, participants were also selected in a non-probabilistic and convenience mode.

#### Inclusion Criteria

- healthy subjects between 18 and 60 years old with previous experience with crutches;
- presenting a normal gait, being asymptomatic on walking at free cadence;
- overcome a simple test of static equilibrium, consisting of keeping one’s balance on each foot for 30 s without great bodily movements [26].

#### Exclusion Criteria

- having an evident disorder of overall coordination and physical skill which could alter the aided gait.

The research protocol was approved by the ethics committee of the University Hospital Virgen Macarena (Seville, Spain). All participants gave written informed consent prior to participation in the study.

### 2.5. Data Collection

**Figure 3:**
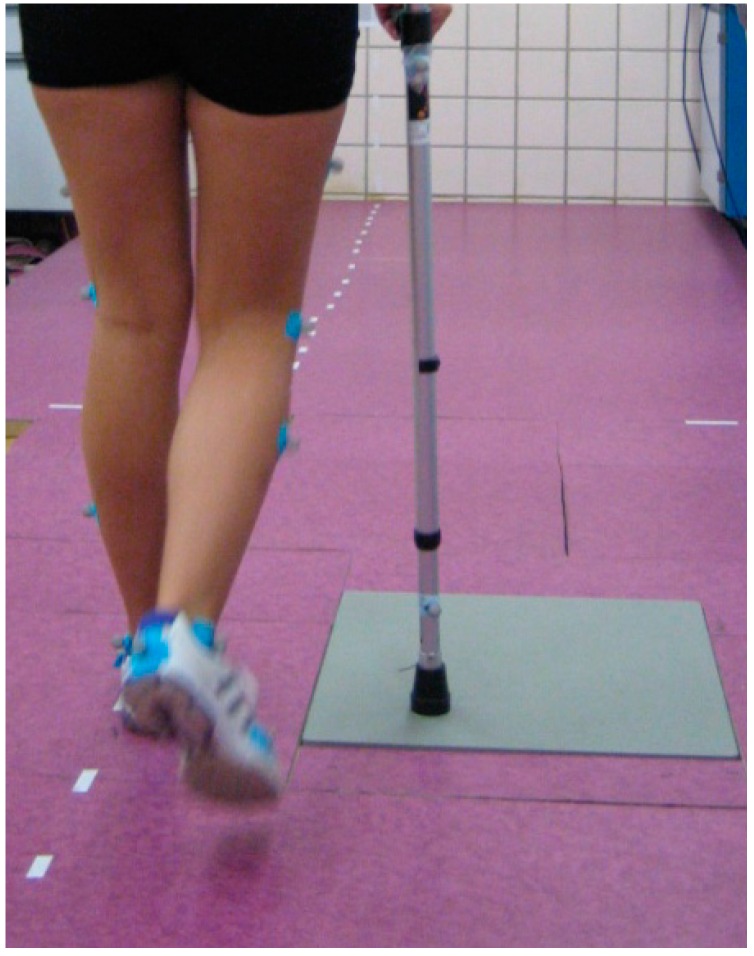
Individual performing aided gait in two stages along the walkway with direction signs and distracting effects so as to avoid him/her focusing on the platform.

The measurements carried out by the two systems during aided gait were taken simultaneously and under the same conditions. The biomechanics laboratory includes a walking corridor 8.5 m long. The force platform was located halfway along the walk. Laterally, and along this corridor, signals were placed in a straight line that offered: the control of the gait direction and a distracting effect that prevented the subjects from centering their attention on the Platform so as to make the crutches match it (Figure 3).

To avoid differences among the tests, the laboratory always used the same artificial light (with lowered blinds) and a constant temperature (25 °C). This data is relevant as the subjects were asked to do physical activity and wear special clothes (sports short, short-sleeved T-shirt and sports shoes).

The participants first had a learning and familiarization period followed by them completing an 8.5 m walk, 10 times with each crutch and with each load percentage (10%, 25% and 50% or maximum possible). Aided gait with a partial load was in two stages, with a contra-lateral crutch and simultaneous heel and crutch support. The chosen height of the crutch was associated with an elbow flexion of 20° to 30° [28,29]. The required speed was at free cadence.

Data collection was performed 10 times with the right crutch and 10 more with the left in each load modality and every patient. Of the valid measurements, we took the three central ones to conduct our statistical analysis. GCH Control Software was used with the GCH System 2.0 and Vicon Nexus (Vicon, Oxford, UK) with the AMTI Platform, controlled by two researchers who recorded the load applied when a crutch matched the Platform. Subsequently, the timing and scoring reference of this support was recorded to correlate the data from both pieces of software in time.

Both measurement systems started at offset or zero, so the weight of the implemented crutch, 0.720 kg, was subtracted from the values of the Platform.

### 2.6. Statistics

The data obtained were organized and analyzed using the IBM SPSS statistical software (Version 22.0; SPSS Inc., Chicago, IL, USA). The descriptive analysis included: mean, standard deviation (SD), minimum, maximum, and percentiles 25, 50 and 75 (P_25_, P_50_ and P_75_). The inferential analysis considered a confidence level of 95%, so that the experimental *p*-value was compared to a significance level of 5%.

To determine the most appropriate test according to data behaviour, we performed the Kolmogorov–Smirnov normality test. According to whether these normality criteria are met or not, the following parametric tests are considered appropriate [30]:

- *t*-test for related samples [30]: it compares the mean values of related samples when the values of the variables meet the normality criteria. This test was used to determine whether the two measurements can be considered similar. In addition, the study was performed based on the different ranges of weight loaded onto the crutches.
- Wilcoxon signed-rank test [30]: it compares the related sample distribution when the values of the variables do not meet the normality criteria.

The intra-class correlation coefficient [31] was used to carry out the concordance analysis between the GCH System and AMTI Platform.

## 3. Results

The descriptive analysis of the variables *Platform* and *GCH* is shown in Table 2 for each crutch and different requested loads (10%, 25% and 50% of body weight). Note that three repeated measures on six different conditions (arm by percentage of load) are taken for every subject.

Table 3 shows the intra-class correlation coefficients (ICCs) for the different values of loads and crutch. Note that ICCs are always between 0.99937 and 0.99995 with *p* < 0.001.

The differences between the average values of AMTI and the right crutch and AMTI and the left crutch were both ≤0.11 kg. In addition, 78.1% of the results (422/540) showed higher values in the Platform than in GCH; in 20.2% (109/540), values were lower and 1.7% (9/540) recorded exactly the same measurement. Although the differences were significant (*p* < 0.01), the global effect-size is 0.028 (and, in all cases, it is always lower than 0.1), so the differences are not relevant from a statistical point of view.

**Figure 4:**
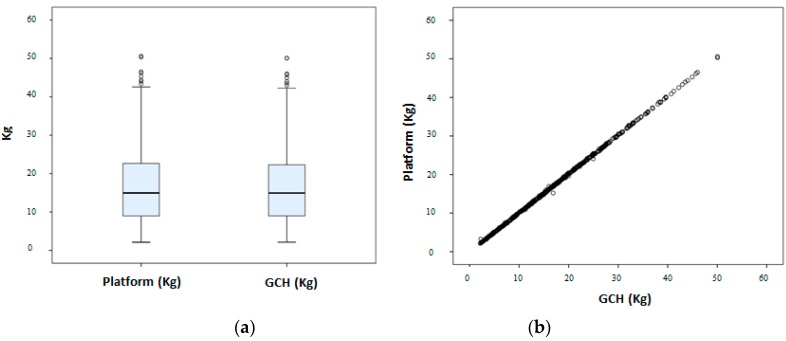
Box plot representing percentiles 25, 50 and 75 (**a**) and scatter plot (**b**) of the relationship between platform and GCH measurements.

Figure 4 shows the similarities between the GCH and Platform measurements.

**Figure 5:**
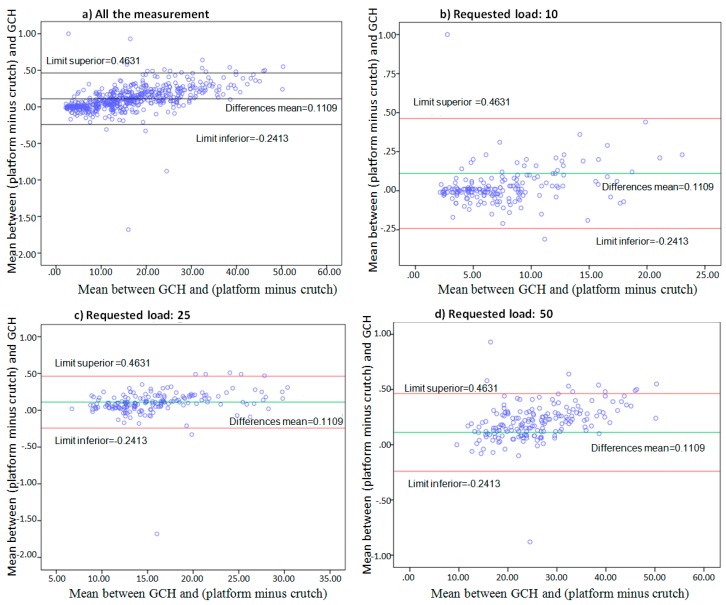
Bland-Altman Method representing the values differences (platform minus crutch) and GCH, versus the means (platform minus crutch) and GCH; for all the measurements (**a**), measurement requesting load on the crutches at 10% of body weight (**b**), 25% (**c**) and 50% (**d**).

The Limits of Agreement (LOA) are shown using a Bland-Altman graphical analysis [32] in Figure 5. The differences between (platform minus crutch) and GCH, versus the means between (platform minus crutch) and GCH, are represented. The study is complemented by Table 4, which determined that the higher the load, the less the number of tolerable measurements. Even so, all of the values were within a 93% tolerance level.

ANOVA, with *p* < 0.001, determined the existence of a linear relationship between both variables. The regression line obtained 99.3% accuracy since the value of the adjusted squared R was 0.993. In the contrast to coefficients, a *p* < 0.05 was obtained, so that both were valid in the regression line. The regression equation obtained was:

*Measurements on Platform = −0.383 + 1.036·Measurements on GCH.* Confidence intervals at 95% for the constant: (−0.519 to −0.247), and for the slope: (1.029 to 1.044).

The breakdown of the regression analysis for each crutch and the different loads is presented in Table 5. Note that the adjusted squared R is in all cases higher than 0.986.

**Figure 6:**
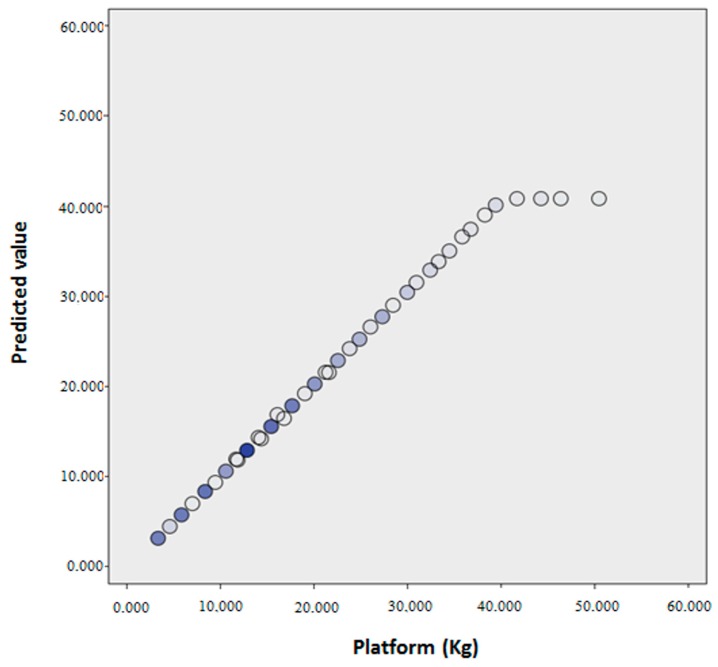
Dispersion graph. Ordenate axis: predicted values by the regression line. Abcissa axis: values recorded by the platform. Color increase in the representation indicates that there are higher values in those in which the variables coincide (0, 10, 20, 30, 40, 50, 60, 70).

Figure 6 shows a comparison between measurements of the Platform and the values obtained through the regression line. The difference between the actual value and the predicted value of the measurements on the Platform was quantified, obtaining an average of 0 (SD = 0.80) and the following percentiles: p25 = −0.12, p50 = 0.03 and p75 = 0.24.

## 4. Discussion

### Validity and Reliability

Intra-class correlation coefficients (ICCs) for the different values of loads and crutches assess the reliability of GCH 2.0 measurements. To validate GCH 2.0, a regression line [33] to determine the predictive ability of the GCH system towards the AMTI Platform was found. In this way, a suitable description of the relationship between both quantitative variables is obtained. The R-squared measure of goodness of fit and the ANOVA of this regression [34] were included in order to check the regression line accuracy and its veracity. Since the force platform is a validated and an effective method to determine the accuracy of the loads, this predictive method is useful as it allows us to use the platform as a reference to validate our system and its measurements. Note that the platform is not a feasible system in gait recovery due to its complexity, difficult handling and high cost [35]. The GCH 2.0 solves these issues for providing effectiveness and efficiency: it is cheaper, requires a minimum space, offers information about load along the way and not only for a single step, the handling is easy, *etc*. Consequently, the instrumented crutch is a better option for gait recovery in patients using forearm crutches.

Regarding the data obtained, the GCH System 2.0 is a valid and reliable tool for assessing the amount of load exerted on the forearm crutches. Consequently, it allows us to quantify said force, providing, at the same time, an objective control of the unloading exerted on the affected limb [13]. Thus, the statistics showed minimum differences between the measurements of the Platform and GCH, interpreting them as non significant. In addition, these non-significant differences were corroborated by conducting comparisons between the Platform and the right crutch as well as between the Platform and the left crutch. Therefore, we conclude that the measurements of both prototypes are equally effective and reproducible.

Secondly, a detailed analysis of these slight disagreements announced that the Platform measurements had a tendency to be higher than those of GCH. This result was expected and assumed by the authors from the beginning of the study, since there are slight mechanical frictions of the outer sleeve that slip over the distal end of the crutch shaft to press the sensor each time a weight is exerted upon it. This friction reduces the GCH values regarding the Platform.

Furthermore, the Bland-Altman plots (Figure 5) show that the greater the load, the greater the differences between measurements. Depending on the different ranges of weight bearing, the difference between measurements of the Platform and GCH increases as weight bearing increases, which is once again expected due to the increase of mechanical friction.

The exhaustive study of the minimum errors due to a mechanical component confirms that they are neither statistically significant nor clinically relevant since a tolerance of up to a kg of error is established [13]. Despite this, the design is more demanding and is aimed at not exceeding 0.5 kg.

This study showed the predictive ability of GCH towards AMTI using a regression line [34]. The mean of the differences between the actual and the predicted value of measurements of the Platform was 0 (SD = 0.80). By comparing the predicted values with the actual values, an almost perfect diagonal between the two variables was achieved, so that it can be confirmed that the predictive model achieved is optimal for research. It is as from 40 kg on the Platform where the predictive model ceases to have a growing tendency, although it is not decisive, as there are very few cases that occur in this range. As noted in Section 3, the differences between the Platform and GCH 2.0 are always too small to be statistically relevant. In all cases, these differences are definitely not clinically relevant.

To sum up, the GCH System 2.0 offers effectiveness in quantifying partial weight-bearing loads applied to the crutches in gait training according to each patient´s particular conditions. Consequently, it is a useful tool to control assisted gait, to obtain accuracy of loads exerted on the crutches and unloadings on the injuried lower limb, to carry out objective progressions [17,36] and to establish new treatment protocols, involving higher quality in the functional gait recovery [37]. Eliminating the subjectivity present in the treatment and raising the accuracy implies a shorter duration and reduction in relapses and sequelae.

As a final comment, according to our experience with healthy subjects with excellent coordinative skills, experience with the use of crutches and training of the exerted loads on the crutches based on feedback, we found that they were unable to control an exact load and maintain it for some time. This conviction is corroborated by Isakov [38], Kaplan [39] and Fu *et al.* [16]. Therefore margins of error of body weight are necessary in order to avoid constant warnings of error by the feedback system. Further work of this paper is the establishment of the ideal margins. In addition, a new study with patients without experience with the use of crutches should be carried out, this being a limitation of this research.

## 5. Conclusions

The GCH System 2.0 constitutes a reliable and valid instrument for measuring weight bearing on implemented forearm crutches during aided gait. The concordance study with the validated AMTI OR6-7-2000 force platform, carried out in assisted gait dynamic conditions, determined the effectiveness of the new prototype in association with the GCH Control Software 1.0 specific program, which allows us to numerically and graphically display loads applied to the crutches through the upper limbs during gait, record them and later analyze them. The System incorporates an optimized acoustic and visual feedback mechanism that increases and maintains the accuracy of the recommended loads for each patient.

As a result, and as opposed to other systems described in literature, our paper presents a compact and ready-to-use device that focuses on accurately monitoring the force applied to crutches during gait, providing personalized feedback to both the patient and the physiotherapist/researcher in order to improve gait training and evaluate the process of functional recovery.

## Figures and Tables

**Table 1:** The main differences between GCH 1.0 and GCH 2.0.

GCH 1.0.	GCH 2.0.
Distributed system	Compact system
Patients have to carry an electronic box place on their belts.	Electronic component inside the crutch tube.
External cables are necessary to connect the sensors to control box placed on the patient´s belt.	Internal cables. Patients do not have any contact with cables.
External electronic components.	Internal miniature electronic components/ surface mount device (SMD).
Weight: 1150 g.	Weight: 720 g.
Non standard battery/rechargeable/700 mA.	Standard battery/AA/rechargeable/6000 mA.
Zero is not automatic.	Offset process is automatically activated.
Only for a patient walking with one or two crutches.	Several patients can use the System simultaneously, with one or two crutches.
Discretized biofeedback. System informs if the load is wrong only with a binary signal.	The physiotherapist/patient can choose between continuous or discretized visual biofeedback. In the continuous mode, the patient receives information throughout the whole process [23].
Moteview 2.0. Generic software that shows: amount of load and a simple linear chart. This is visualized by the researcher. It is not useful for the patient.	GCH Control Software 1.0.: Specific program to control assisted gait. The load could be shown in percentages of the patient´s weight-bearing (data of clinic interest). It offers specific charts and data for researchers, physiotherapists and patients. It is adaptable to the kind of patient. (Figure 2).
No database.	Patients’ clinical database.
Data sampling frequency ≤10 Hz	Data sampling frequency ≤80 Hz
The portable system. The physiotherapist selects the ideal load without percentages. It does not allow for comparisons and research.	The portable system (watch). The physiotherapists or researchers select the ideal load or the percentage of the patient’s weight-bearing (data of clinic interest).

**Table 2:** Descriptive analysis of the Platform and GCH for each crutch and different loads.

		Load	N	Mean*	SD	Minimum	Maximum	Percentiles		
								25	50	75
**Platform**	**Right crutch**	10	90	7.33	4.00	2.24	20.09	4.38	6.37	9.00
		25	90	16.03	4.86	9.07	29.89	12.44	15.04	19.00
		50	90	25.66	8.45	12.64	50.60	19.33	24.23	30.63
	**Left crutch**	10	90	7.76	4.30	2.13	23.15	4.58	6.73	9.33
		25	90	15.79	5.07	6.72	30.50	12.64	14.89	17.42
		50	90	26.48	8.28	9.58	46.52	20.12	25.45	32.59
**GCH**	**Right crutch**	10	90	7.31	3.97	2.20	19.65	4.40	6.30	9.07
		25	90	15.93	4.82	8.99	29.73	12.42	14.95	18.90
		50	90	25.46	8.38	12.70	50.06	19.02	24.38	30.40
	**Left crutch**	10	90	7.73	4.26	2.14	22.92	4.57	6.76	9.25
		25	90	15.69	5.02	6.70	30.19	12.56	14.77	17.32
		50	90	26.27	8.22	9.58	46.02	19.88	25.23	32.17

* Values are presented in Kg.

**Table 3:** Intra-class correlation coefficients between Platform and GCH.

	Load	Intra-Class Correlation	Confidence Interval (95%)		*p*-Value
			Lower Bound	Upper Bound	
**Right crutch**	10	0.99964	0.99946	0.99976	<0.001
	25	0.99937	0.99904	0.99958	<0.001
	50	0.99985	0.99977	0.99990	<0.001
**Left crutch**	10	0.99990	0.99985	0.99994	<0.001
	25	0.99993	0.99990	0.99996	<0.001
	50	0.99995	0.99992	0.99996	<0.001
**Global**		**0.99992**	**0.99990**	**0.99993**	**<0.001**

**Table 4:** Frequencies and load percentages of adjustment for the tolerance levels according to the Bland–Altman method. (*n* = number of measurements).

Requested Load	Tolerance Level	*n*	%
**10%**	Tolerable	178	98.9
	Not tolerable	2	1.1
**25%**	Tolerable	173	96.1
	Not tolerable	7	3.9
**50%**	Tolerable	169	93.9
	Not tolerable	11	6.1
**Global**	**Tolerable**	**520**	**96.3**
	**Not tolerable**	**20**	**3.7**

**Table 5:** Regression analysis for each crutch and the different loads.

	Right Crutch			Left Crutch			Global
Load	10	25	50	10	25	50	
**Constant**	−0.072	−0.023	−0.016	−0.369	−0.045	0.009	**−0.383**
Constant Lower Bound	−0.167	−0.198	−0.146	−0.598	−0.093	−0.066	**−0.519**
Constant Upper Bound	0.024	0.153	0.115	−0.140	0.003	0.083	**−0.247**
Constant *p*-value	0.138	0.799	0.809	0.002	0.067	0.813	**<0.001**
**Slope**	1.014	1.008	1.009	1.062	1.009	1.008	**1.036**
Slope Lower Bound	1.003	0.997	1.004	1.036	1.006	1.005	**1.029**
Slope Upper Bound	1.026	1.018	1.013	1.089	1.012	1.010	**1.044**
Slope *p*-value	<0.001	<0.001	<0.001	<0.001	<0.001	<0.001	**<0.001**
**Adjusted Squared R**	0.997	0.998	0.999	0.986	>0.999	>0.999	**0.993**
Regression *p*-value	<0.001	<0.001	<0.001	<0.001	<0.001	<0.001	**<0.001**

## Abbreviations

The following abbreviations are used in this manuscript:

ANOVA	Analysis of Variance
FS	Full Scale
PCB	Printed Circuit Board
PPBW	Percentage of Patient Body Weight
SD	Standard Deviation
SMD	Surface Mount Device
